# Loss of Nuclear Activity of the FBXO7 Protein in Patients with Parkinsonian-Pyramidal Syndrome (PARK15)

**DOI:** 10.1371/journal.pone.0016983

**Published:** 2011-02-11

**Authors:** Tianna Zhao, Esther De Graaff, Guido J. Breedveld, Agnese Loda, Lies-Anne Severijnen, Cokkie H. Wouters, Frans W. Verheijen, Marieke C. J. Dekker, Pasquale Montagna, Rob Willemsen, Ben A. Oostra, Vincenzo Bonifati

**Affiliations:** 1 Department of Clinical Genetics, Erasmus MC, Rotterdam, The Netherlands; 2 Department of Neurology, Radboud University Medical Center, Nijmegen, The Netherlands; 3 Department of Neurology, University of Bologna, Bologna, Italy; French National Centre for Scientific Research, France

## Abstract

Mutations in the *F-box only protein 7* gene (*FBXO7*) cause PARK15, an autosomal recessive neurodegenerative disease presenting with severe levodopa-responsive parkinsonism and pyramidal disturbances. Understanding the PARK15 pathogenesis might thus provide clues on the mechanisms of maintenance of brain dopaminergic neurons, the same which are lost in Parkinson's disease. The protein(s) encoded by *FBXO7* remain very poorly characterized. Here, we show that two protein isoforms are expressed from the *FBXO7* gene in normal human cells. The isoform 1 is more abundant, particularly in primary skin fibroblasts. Both isoforms are undetectable in cell lines from the PARK15 patient of an Italian family; the isoform 1 is undetectable and the isoform 2 is severely decreased in the patients from a Dutch PARK15 family. In human cell lines and mouse primary neurons, the endogenous or over-expressed, wild type FBXO7 isoform 1 displays mostly a diffuse nuclear localization. An intact N-terminus is needed for the nuclear FBXO7 localization, as N-terminal modification by PARK15-linked missense mutation, or N-terminus tag leads to cytoplasmic mislocalization. Furthermore, the N-terminus of wild type FBXO7 (but not of mutant FBXO7) is able to confer nuclear localization to profilin (a cytoplasmic protein). Our data also suggest that overexpressed mutant FBXO7 proteins (T22M, R378G and R498X) have decreased stability compared to their wild type counterpart. In human brain, FBXO7 immunoreactivity was highest in the nuclei of neurons throughout the cerebral cortex, intermediate in the globus pallidum and the substantia nigra, and lowest in the hippocampus and cerebellum. In conclusion, the common cellular abnormality found in the PARK15 patients from the Dutch and Italian families is the depletion of the FBXO7 isoform 1, which normally localizes in the cell nucleus. The activity of FBXO7 in the nucleus appears therefore crucial for the maintenance of brain neurons and the pathogenesis of PARK15.

## Introduction

Parkinson's disease (PD), the second most common neurodegenerative disorder after Alzheimer's disease, is pathologically characterized by the progressive loss of dopaminergic neurons in the substantia nigra of the midbrain, and the formation of alpha-synuclein-containing protein aggregates, termed Lewy bodies, in surviving neurons [1]. In recent years, defective ubiquitin-proteasome system, mitochondrial dysfunction, oxidative stress, and autophagy impairment have all been suggested to play some roles in the PD pathogenesis, but the primary molecular mechanisms of this disease remain mostly unknown [2], [3]. PD is a sporadic, idiopathic disorder in most patients, but the identification of genetic mutations causing rare Mendelian forms of parkinsonism has provided novel clues for understanding of the disease pathogenesis [3], [4]. Some of these Mendelian parkinsonism, such as those caused by dominant mutations in the *alpha-synuclein* (PARK1) or *leucine-rich repeat kinase 2* (PARK8) gene, are more similar to the common, idiopathic PD form [5]. In other forms, such as those caused by recessive mutations in the *parkin* (PARK2), *PINK1* (PARK6), *DJ-1* (PARK7), and *ATP13A2* (PARK9), the phenotype is more often atypical due to younger-onset, presence of additional clinical signs (dementia, pyramidal signs), or absence of Lewy body-pathology [6], [7], [8]. However, despite these atypical phenotypes, understanding the mechanisms of the Mendelian parkinsonisms might provide important clues into the pathways leading to the degeneration of the dopaminergic neurons, which might also be involved in the common forms of PD. For example, the ATP13A2 protein has been recently identified as a potent modifier of the toxicity induced by alpha-synuclein in animal models of PD [9].

Recently, we characterized mutations in the *F-box only protein 7* (*FBXO7*) gene, encoding the F-box protein 7 (FBXO7), as the cause of PARK15, an autosomal recessive neurodegenerative disease presenting with juvenile, severe levodopa-responsive parkinsonism and additional pyramidal signs [10]. A homozygous *FBXO7* nonsense mutation (R498X) is present in an Italian family, while compound heterozygous mutations (IVS7+1G/T and a T22M mutation) are found in a Dutch family. Another homozygous mutation (R378G) was previously identified by others in an Iranian family [11].

FBXO7 is a member of the F-box-containing protein (FBP) family, characterized by a ∼40-amino acids domain (the F-box) [12], [13], [14]. FBPs serve as molecular scaffolds in the formation of protein complexes, and have been implicated in a range of processes, such as cell cycle, genome stability, development, synapse formation, and circadian rhythms (reviewed in [15]). Through the interaction between the F-box and the Skp1 protein, FBPs might become part of SCF (Skp1, Cullin1, F-box protein) ubiquitin ligase complexes, and play roles in ubiquitin-mediated proteasomal degradation [15]. However, FBPs might also be involved in ubiquitin-mediated, non-proteasomal pathways, and SCF-independent non-proteolytic functions [15].

Two *FBXO7* transcript variants are annotated in Genbank (accession number NM_012179.3 and NM_001033024.1), resulting from the usage of different open reading frame (ORF) start codons on alternatively spliced 5′-exons, and predicted to encode two FBXO7 protein isoforms of 522 and 443 amino acids (also referred to as isoform 1 and isoform 2). However, the experimental confirmation of the existence of these two protein isoforms has remained elusive. Nothing is known about the expression of the FBXO7 protein(s) in the human brain.

**Figure 1:**
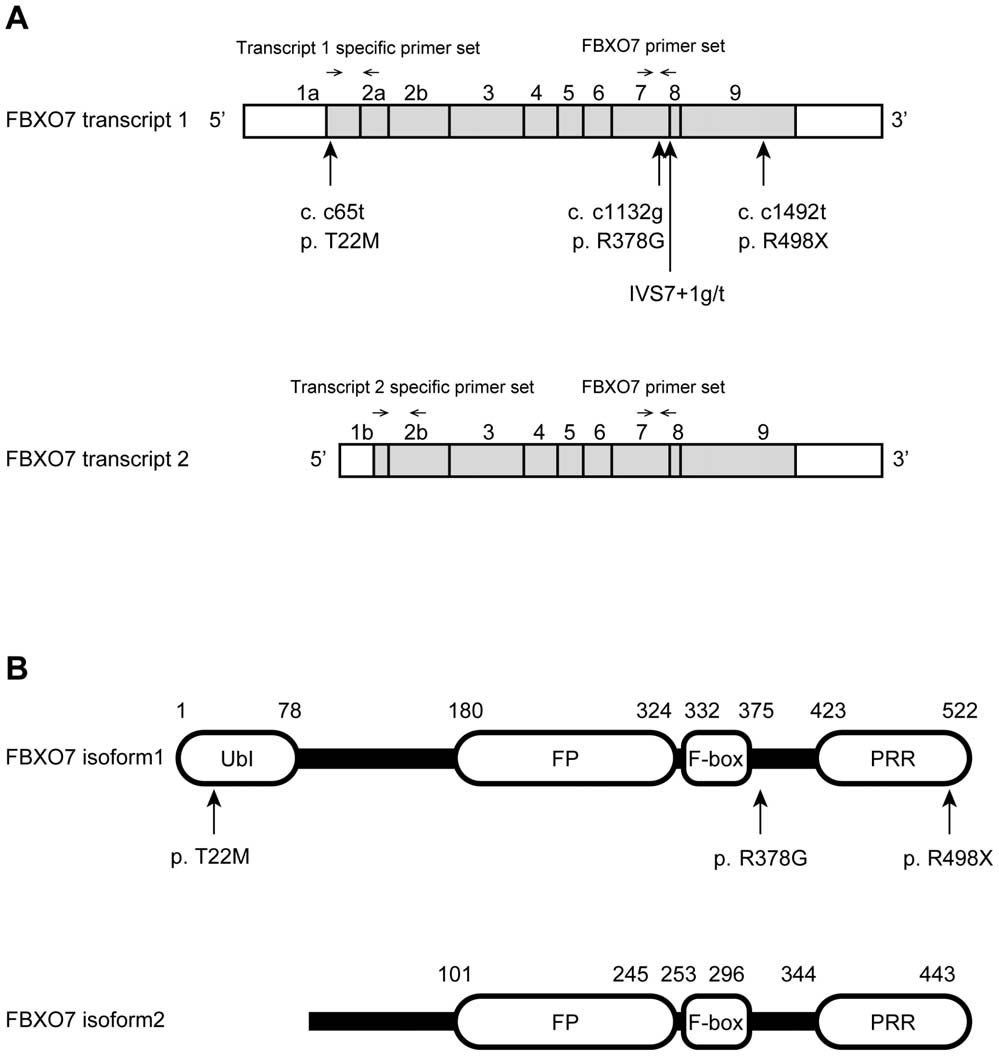
Schematic representation of the *FBXO7* transcripts and protein isoforms. (A) The *FBXO7* transcripts with location of the mutations found in patients with PARK15. The location of the primers used in qPCR is indicated by arrows. (B) Domain organization of the two FBXO7 protein isoforms. Ubl: ubiquitin-like domain; FP: FBXO7/ PI31 domain; F-box: F-box motif; PRR: proline rich domain.

The FBXO7 isoform 1 is longer than isoform 2 because of the presence of an N-terminal ubiquitin-like (Ubl) domain (absent in the isoform 2), which is thought to interact with ubiquitin receptor proteins (Figure 1). The remaining domains are present in both isoforms, including an FP (FBXO7/PI31) domain, the F-box motif, and a C-terminal proline-rich region (PRR). The FP domain mediates the interaction of FBXO7 with the proteasome inhibitor protein PI31 [16]. The PRR appears crucial for the binding of FBXO7 to its reported substrates.

Very little is known about the function and the sub-cellular localization of these two FBXO7 proteins. FBXO7 has been reported to interact with the hepatoma up-regulated protein (HURP, a mitotic protein) [17], the inhibitor of apoptosis protein 1 (cIAP1) [18], and the proteasome inhibitor protein PI31 [16]. Last, FBXO7 was shown to possess SCF-independent transforming activity by enhancing the interaction of cyclin-dependent-kinase CDK6 with its targets [19]. Whether these or other still unknown FBXO7 interacting-proteins are important for the neuronal function of FBXO7 and for the mechanisms of neurodegeneration remains unknown. Here, we show the existence of two FBXO7 protein isoforms in normal human cells; we characterize their subcellular localization and their differential depletion in cell lines from the patients with PARK15; last, we characterize the expression of the FBXO7 proteins in the normal human brain.

## Results

### Expression of the FBXO7 proteins in families with PARK15

To study the expression of the endogenous FBXO7 proteins, we obtained Epstein-Barr virus (EBV)-transformed lymphoblastoid cell lines from members of the Italian and Dutch PARK15 families, and from unrelated normal controls. Fibroblast lines were also established from skin biopsies in one Dutch PARK15 patient and one unrelated control. Unfortunately, fibroblasts from the second Dutch PARK15 patient and from patients of the Italian PARK15 family were not available.

**Figure 2:**
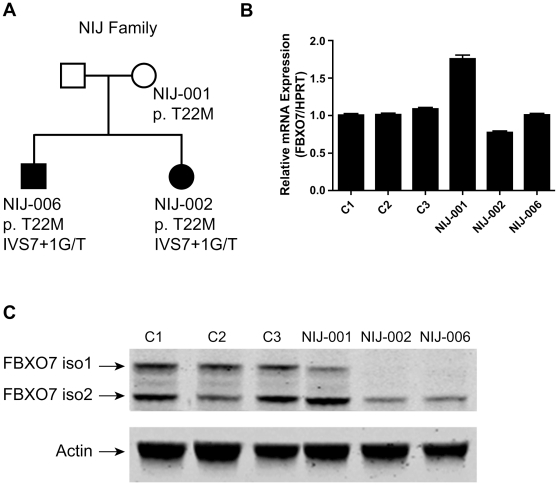
Expression of FBXO7 in the Dutch PARK15 family. (A) Family pedigree and *FBXO7* genotypes. (B) qPCR analysis of *FBXO7* mRNA (both transcript isoforms) in members of the PARK15 family and unrelated, healthy controls (C1–C3). (C) Western blotting analysis. The two FBXO7 isoforms present in controls are altered in the mutation carriers. The isoform 1 is undetectable in the patients (NIJ-002 and NIJ-006) and decreased in the mother (NIJ-001), who also carries the mutation affecting this isoform (T22M). The isoform 2 is decreased in the patients and normal in the mother (see text for further details).

**Figure 3:**
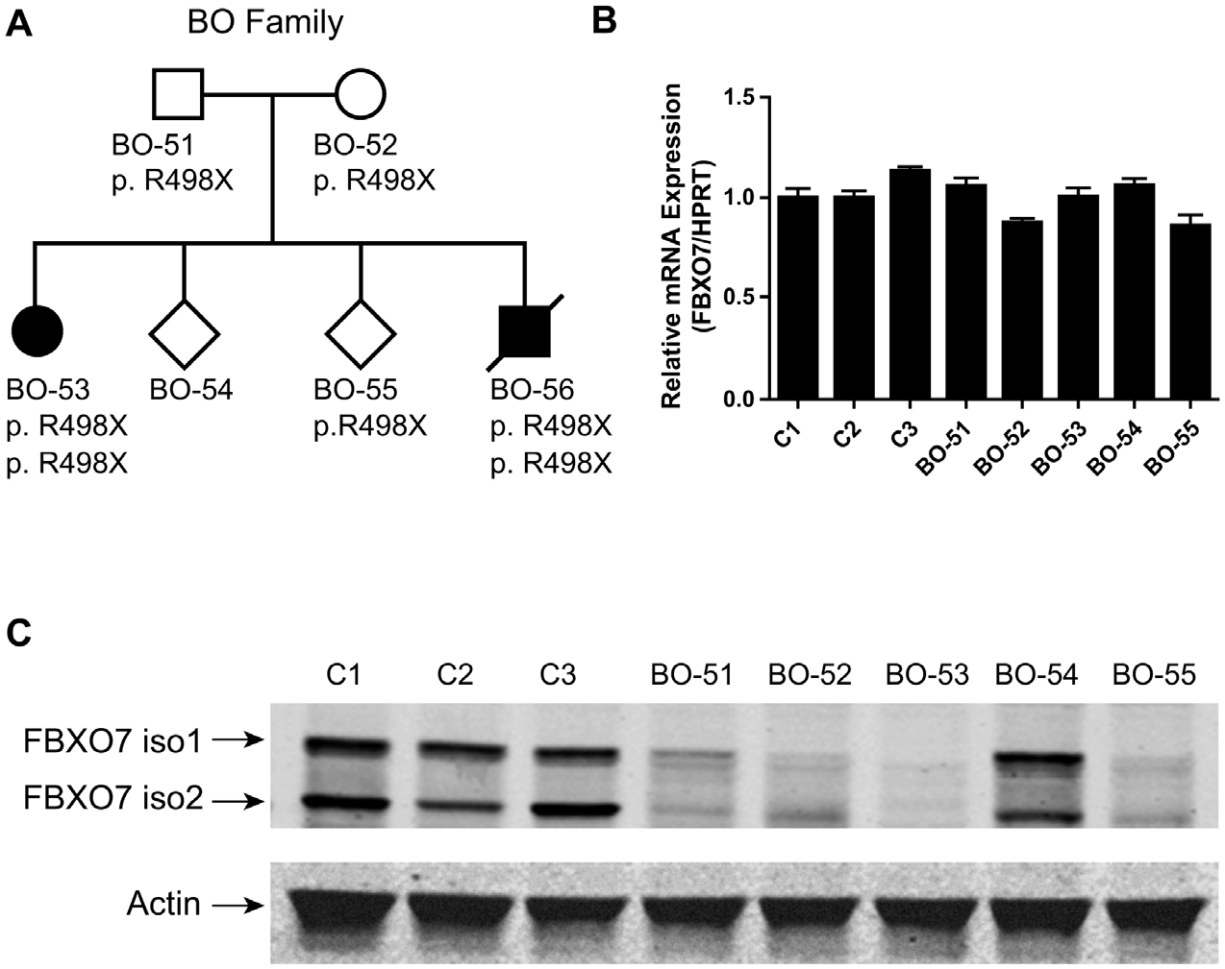
Expression of FBXO7 in the Italian PARK15 family. (A) Family pedigree and *FBXO7* genotypes. (B) qPCR analysis of *FBXO7* mRNA (both transcript isoforms) in members of the PARK15 family and unrelated, healthy controls (C1–C3). (C) Western blotting analysis. The two FBXO7 isoforms present in controls are both undetectable in the PARK15 patient (BO-53), and markedly decreased in the heterozygous mutation carriers (BO-51, BO-52, BO-55). The BO-54 subject is not a carrier of the mutation and shows normal FBXO7 expression.

In lymphoblastoid cells from normal controls (Figures 2 and 3), and HEK 293T cells (Figure S1), our Western blot (WB) analysis using an antibody against FBXO7 revealed two bands of the expected molecular weight of the FBXO7 isoform 1 and isoform 2. The knock down (KD) of *FBXO7* gene in HEK 293T cells confirmed the specificity of the antibody for the FBXO7 proteins (Figure S1).

In the Dutch family, two siblings were affected by PARK15 and they carried compound heterozygous *FBXO7* mutations: the splice-site mutation IVS7+1G/T, which removes the invariable splice donor of intron 7 and is expected to disrupt the splicing of both the *FBXO7* transcript isoforms; and a substitution in exon 1A, c.C65T, predicted to lead to the missense change p.T22M, but only in the longer FBXO7 protein isoform 1 (Figure 2A). We previously documented the expression of both *FBXO7* alleles in these patients, by verifying the presence of the heterozygous c.C65T mutation in cDNA from blood cells [10]. We also showed multiple aberrantly spliced transcripts resulting from the activation of cryptic splice sites in exon 7, and encoding frame-shift proteins followed by premature truncation [10]. These are usually unstable and rapidly degraded by the cell.

In the Dutch PARK15 patients, the FBXO7 isoform 1 was undetectable in WB, while the isoform 2 was detected in lower amounts (Figure 2C). The unaffected mother (NIJ-001) who only carried the T22M mutation, showed lower amount of the isoform 1, and normal amount of isoform 2. These results are compatible with lack of FBXO7 protein expression from the allele containing the IVS7+1G/T mutation, and with only isoform 2 being expressed from the allele containing the T22M mutation. In other words, the T22M mutation leads to selective depletion of the isoform 1, the only isoform in which this mutation is incorporated.

To exclude effects of these mutations at mRNA level, or presence of other, unknown mutations in linkage disequilibrium, we performed quantitative PCR (qPCR) analysis of the total FBXO7 transcripts, as well as the isoform 1-specific and the isoform 2-specific transcripts separately. These experiments showed that the *FBXO7* mRNA levels in the Dutch patients were similar to those in unrelated controls (Figure 2B and Figure S3), suggesting that the main effect of these mutations is at the level of protein stability.

The two affected siblings in the Italian PARK15 family carry an *FBXO7* homozygous nonsense mutation in exon 9, predicted to affect both transcripts (c.C1492T, according to the longer *FBXO7* transcript, protein effect p.R498X) (Figure 3A). qPCR analysis showed similar *FBXO7* mRNA levels among unrelated healthy controls, R498X heterozygous and homozygous mutation carriers (Figure 3B and Figure S3), indicating that this truncating mutation escapes nonsense-mediated mRNA decay [20]. However, in WB analysis, the R498X heterozygous carriers displayed reduced levels of both the FBXO7 protein isoforms, while the FBXO7 proteins were both undetectable in the homozygous PARK15 patient (Figure 3C). Unfortunately, the second patient in this family died before cell lines could be obtained. Thus, in the case of this nonsense mutation, the main final effect is the depletion of both FBXO7 protein isoforms, likely due to protein instability. Since this mutation only removes the last 24 residues of FBXO7, the C-terminus appears therefore very important for the stability of this protein.

### Subcellular localization of wild type and mutant FBXO7

**Figure 4:**
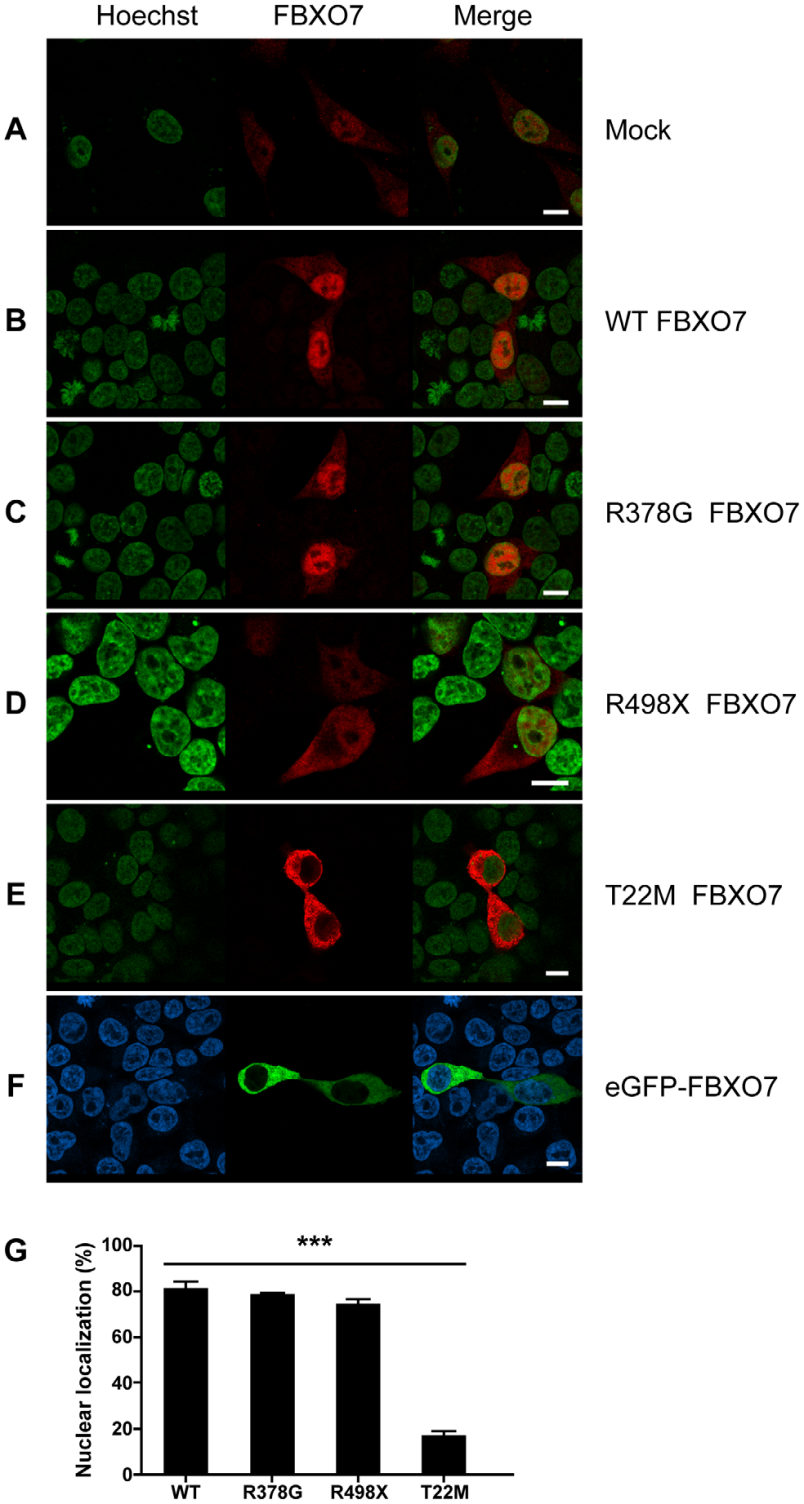
Localization of FBXO7 in HEK 293T cells. Cells were transfected with empty vector (mock, A), wild type *FBXO7* (B), R378G mutant (C), R498X mutant (D), T22M mutant (E) and N-terminus-tagged *eGFP*-*FBXO7* (F). In panels A–E, the FBXO7 protein is visualized in red by using a mouse primary anti-FBXO7 antibody and a Cy3-coupled secondary anti-mouse antibody. In panel F, the FBXO7 protein is directly visualized by the green eGFP signal. The nucleus (Hoechst staining) is depicted in green, with the exception of panel F, where it is stained in blue (scale bars, 10 µm). (G) Quantification of the nuclear localization of FBXO7. At least 200 HEK 293T cells expressing FBXO7 were counted. *** *p*<0.01 (chi-square test - T22M versus wild type and other FBXO7 variants).

To investigate the subcellular localization of FBXO7, we transiently transfected HEK 293T cells with plasmids overexpressing the wild type (WT) or mutant FBXO7 isoform 1. Forty-eight hours after transfection we analyzed the cells by immunofluorescence and confocal microscopy. In mock transfected HEK 293T cells, a main nuclear and much weaker cytosolic staining was observed (Figure 4A). *FBXO7* stable knock-down in these cells confirmed the specificity of the staining, which represents therefore the endogenous FBXO7 protein (Figure S2). The overexpression of the WT FBXO7 isoform 1 in HEK 293T cells resulted in a mostly nuclear staining, a pattern shared by the R378G mutant (Figure 4B and Figure 4C). A much weaker signal was still diffusely detected in the cytoplasm.

**Figure 5:**
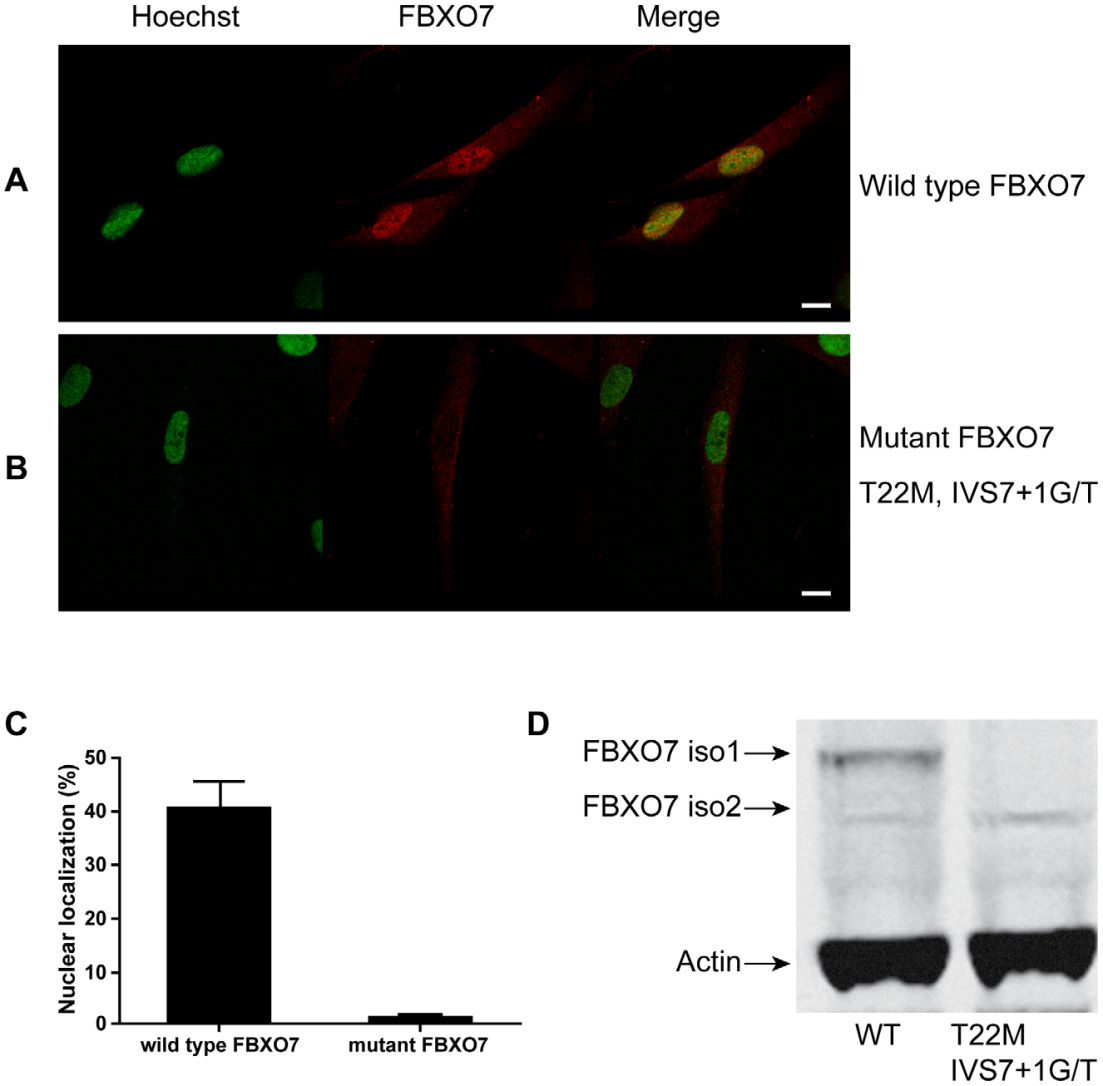
Expression of endogenous FBXO7 in human fibroblasts. (A, B) For immunofluorescence, the FBXO7 protein is visualized in red by using a mouse primary anti-FBXO7 antibody and a Cy3-coupled secondary anti-mouse antibody. The nucleus (Hoechst staining) is depicted in green. (scale bars, 10 µm). (A) normal control; (B) Dutch PARK15 patient with T22M and IVS7+1G/T mutations. (C) quantification of percentages of cells showing mainly nuclear localization of FBXO7. (D) Western blotting analysis of fibroblasts from a normal control and the Dutch PARK15 patient.

To confirm the localization of endogenous wild type and mutant FBXO7, we performed immunofluorescence in fibroblasts from one Dutch PARK15 patient, and one unrelated normal control (Figure 5). A pattern of mainly nuclear fluorescence (similar to that seen in HEK 293T cells in Figure 4A) was often observed in the control fibroblasts, but never in the patient fibroblasts (Figure 5). WB analysis confirmed the expression of two FBXO7 isoforms in normal fibroblasts (Figure 5D); compared with lymphoblasts, here the isoform 1 was much more abundant than isoform 2. In the fibroblasts from the Dutch PARK15 patient, the isoform 1 was undetectable by WB, while isoform 2 was detected, in agreement with the results of WB in lymphoblastoid cells from the same patient (Figure 2C). Taken together, the experiments in the patients fibroblasts show that the loss of isoform 1 (WB) is associated with the loss of immunoreactivity in the nucleus (seen in immunofluorescence). The weak cytosolic immunoreactivity is compatible with the residual expression of isoform 2 seen in this patient in WB.

**Figure 6:**
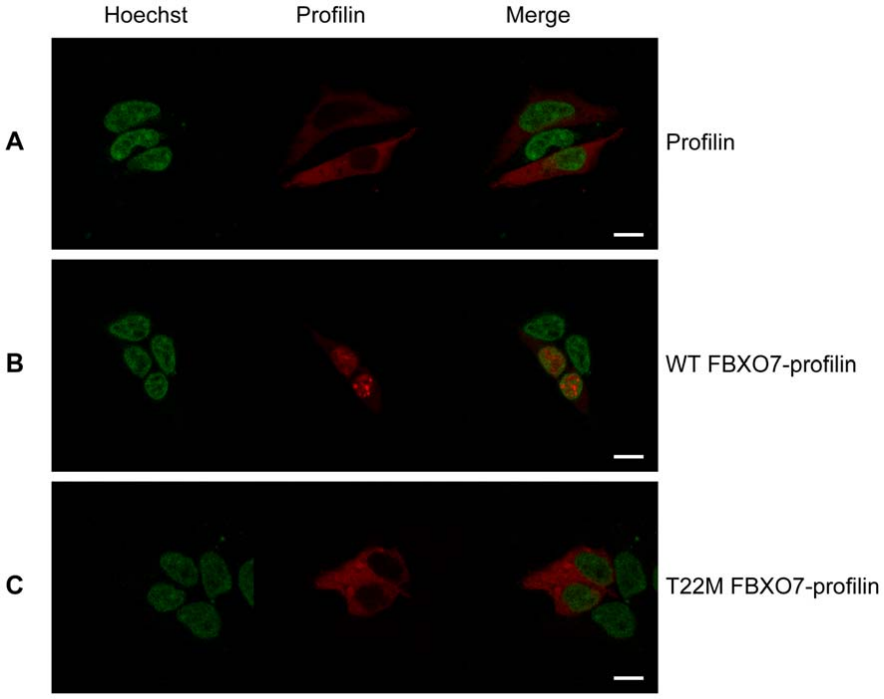
The N-terminus of FBXO7 confers nuclear localization to profilin. In panel (A), Profilin, a well-known cytosolic protein, is visualized by the red mCherry signal; the nucleus is depicted in green (Hoechst staining). Expressing the first 40 amino acids of WT-FBXO7 isoform 1 in front of mCherry-labeled profilin changes the localization of profilin from the cytoplasm to the nucleus (B). The same FBXO7 N-terminal peptide carrying the T22M-mutation found in PARK15 patients is totally devoid of this capacity (C). (scale bars, 10 µm).

The FBXO7 R498X mutant displayed an abnormal pattern consisting of diffuse nuclear and cytosolic localization when overexpressed in HEK 293T cells (Figure 4D). Last, the T22M mutant showed the most striking aberrant pattern of mostly cytosolic localization (Figure 4E and Figure 4G). The T22M mutation is close to the N-terminus where it might impair a nuclear localization signal. To test this hypothesis, we overexpressed WT FBXO7 with an N-terminal tag (enhanced green fluorescent protein, eGFP). As expected, the N-terminal tagging totally blocked the nuclear localization of the protein (Figure 4F), mimicking the pattern of the T22M mutant. Furthermore, the first 40 amino acids of WT or T22M-mutant FBXO7 isoform 1 were cloned in front of the mCherry-labeled profilin, a well-known cytosolic protein. As a result, the WT FBXO7 N-terminal 40 amino acid peptide, but not the T22M-mutant, was able to change the localization of profilin from cytoplasm to nucleus (Figure 6).

**Figure 7:**
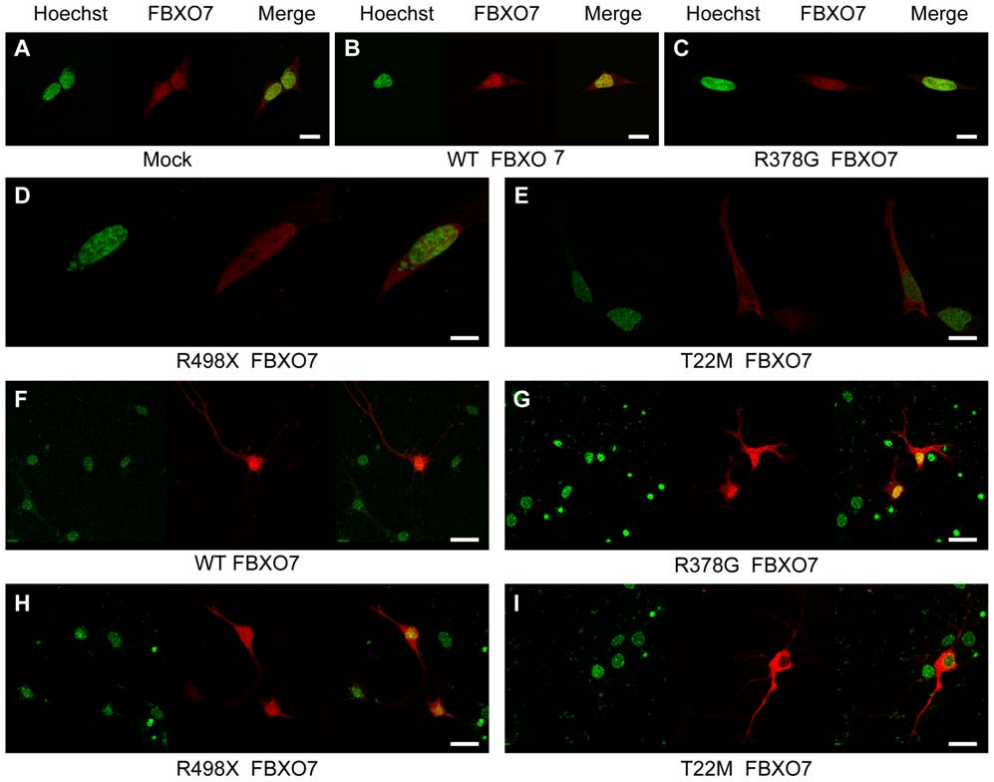
Localization of FBXO7 in SH-SY5Y cells and primary hippocampal neurons. Neuroblastoma SH-SY5Y cells (A–E) and mouse primary hippocampal neurons (F–I) were transfected with empty vector (mock, A), wild type FBXO7 (B, F), R378G mutant (C, G), R498X mutant (D, H), and T22M mutant (E, I) FBXO7. The FBXO7 protein is visualized in red by using a mouse primary anti-FBXO7 antibody and a Cy3-coupled secondary anti-mouse antibody. The nucleus (Hoechst staining) is depicted in green. (scale bars, 20 µm).

The localization pattern of WT- and mutant FBXO7 proteins in human neuroblastoma SH-SY5Y cells and mouse primary hippocampal neurons were similar to those described in HEK 293T cells (Figure 7A–I).

### Stability of mutant FBXO7 *in vitro*

On the basis of the WB and qPCR observations on the cells from the PARK15 patients, we hypothesized that the missense and nonsense mutants (T22M and R498X) markedly decreased the stability of FBXO7.

**Figure 8:**
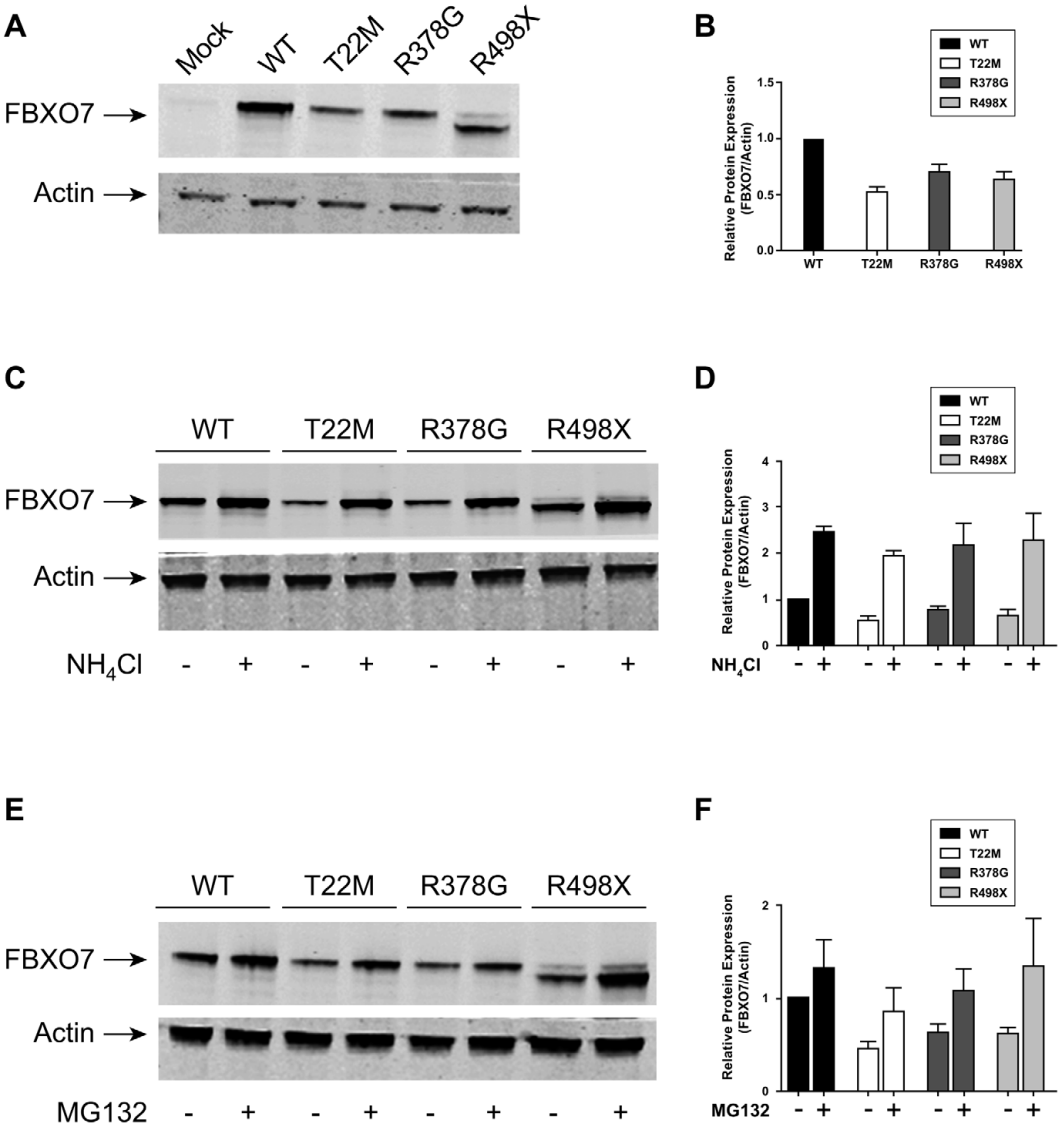
Overexpression of wild type and mutant FBXO7. Overexpression of wild type and mutant FBXO7 proteins in HEK 293T cells (A), and effect of the treatment with the lysosomal inhibitor NH_4_Cl (C) and the proteasomal inhibitor MG-132 (E). Actin is used as loading control. The quantification of the protein levels is shown in the right panels (B, D, and F) (Odyssey software).

To test this hypothesis, the WT and mutant *FBXO7* (T22M and R498X) were transfected in HEK 293T cells. The R378G mutant reported previously in an Iranian PARK15 family [11] was also tested. The overexpression of all these FBXO7 mutants yielded consistently and significantly decreased levels in WB compared with their WT counterpart (Figure 8A–B). This is consistent with our observations in the patients cells and suggest that the FBXO7 mutants are rapidly degraded. To explore the degradation pathways of FBXO7, the lysosomal inhibitor NH_4_Cl or the proteasome inhibitor MG-132 were added to the cells overexpressing WT and mutant FBXO7. Treatment with NH_4_Cl strongly inhibited the degradation of FBXO7, increasing the steady-state protein expression by three to four fold (Figure 8C–D), which suggests that overexpressed FBXO7 is mainly degraded by the lysosomal pathway. The treatment with MG-132 leads to smaller increases in steady-state FBXO7 expression (Figure 8E–F), indicating a minor role of the proteasome degradation pathway. To control for the transfection efficiency, the WT and mutant *FBXO7* (T22M, R378G, and R498X) were transfected in HEK 293T cells together with the eGFP protein. Similar results were obtained (Figure S4).

### FBXO7 proteins expression in the human brain

**Figure 9:**
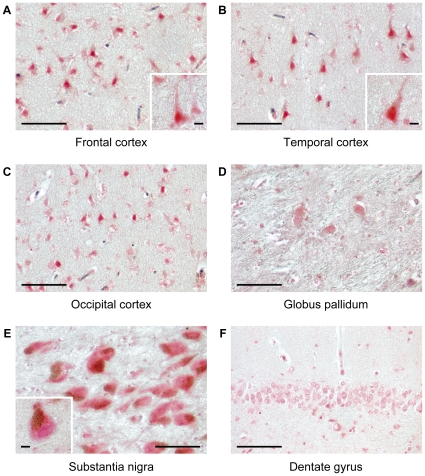
Expression of the FBXO7 protein in the normal human brain. Brain sections are stained using anti-FBXO7 antibody (Abnova): frontal (A), temporal (B), and occipital (C) cortex; globus pallidum (D); substantia nigra (E); dentate gyrus of hippocampus (F). (scale bars, 100 µm).

To characterize the expression of the FBXO7 proteins in the human brain, we studied the following brain regions by immunohistochemistry: frontal, temporal, and occipital cerebral cortex, hippocampus, globus pallidum, substantia nigra, and cerebellar cortex. The immunoreactivity was highest in the nuclei of neurons throughout the cerebral cortex, intermediate in neurons in the globus pallidum and the substantia nigra, and lowest in the hippocampus (Figure 9) and cerebellar cortex (not shown).

## Discussion

Recessive *FBXO7* mutations are definitely established as the cause of PARK15, a novel form of juvenile neurodegenerative parkinsonism with additional pyramidal signs [10], [11]. However, very little is known about the biology of the FBXO7 proteins. Even the existence of the two protein isoforms remained to be confirmed, the subcellular localization was poorly characterized, and the expression in the human brain unexplored. A localization of overexpressed FBXO7 to the cytoplasm and nucleus has been reported in previous studies which all used N-terminal tags to visualize FBXO7 (mostly isoform 1), and did not control for tag-related effects [16], [18], [19]. The N-terminal tagging was probably responsible for the observed cytosolic localization of FBXO7 in those studies. The localization of the over-expressed untagged FBXO7 or of the endogenous FBXO7 was not previously investigated. This uncertainty in the subcellular localization of the FBXO7 protein also complicates the interpretation of the biological plausibility of the reported interactions with other proteins. On the other hand, it is crucial to assess whether the FBXO7 disease-causing mutations affect protein stability, before these mutants are used in functional studies and conclusions are made about possible pathogenetic mechanisms.

A first important message of this study is that two FBXO7 protein isoforms are expressed in normal human cells. This contention is supported by the fact that the two FBXO7 immunoreactive bands are both undetectable in HEK 293T cells with stable *FBXO7* gene KD, and in the cells from the patients with PARK15 who carry a homozygous truncating mutation predicted to affect both the *FBXO7* transcripts. In keeping with this model, only the FBXO7 isoform 1 is markedly depleted in the Dutch patients, as one of the mutations present in those patients (T22M) is indeed only affecting the isoform 1, leaving the isoform 2 unaffected. Thus, the Dutch patients are still able to express the isoform 2 from this mutant allele, while the other allele (carrying the IVS7+1G/T splice mutation) disrupts the expression of both isoforms. Interestingly, the heterozygous mutation carriers of the Italian family, who display lower levels of both FBXO7 isoforms, remain healthy (Figure 3). On the contrary, the Dutch patients, who still express low levels of only the isoform 2, are affected (Figure 2). This strongly suggests that the depletion of the isoform 1 is the culprit for the pathogenesis of PARK15.

Furthermore, we show that the overexpressed, untagged FBXO7 isoform 1 and also the endogenous FBXO7 protein are mostly localized to the cell nucleus in two human cell models (HEK 293T and SH-SY5Y), as well as in mouse primary hippocampal neurons and human skin fibroblasts. We also show that an intact N-terminus is essential for proper nuclear localization of FBXO7 isoform 1. Modifications in this region of FBXO7, including the missense T22M mutation or N-terminal tagging by eGFP, lead to mislocalization to the cytoplasm. These observations also support the contention that previous reports of cytoplasmic localization of FBXO7 are probably due to N-terminal tagging.

Canonical nuclear localization signals are not present in the N-terminus of FBXO7. However, nucleus localization signals might also consist of one or more short sequences of positively charged lysine or arginine residues, which are indeed present in the N-terminus of FBXO7 (Figure S5) [21], [22]. The effects of N-terminal tagging would therefore be explained by the masking of these signals. How the T22M mutation prevents the nuclear localization of overexpressed FBXO7 is unclear, but the mutation might affect the interaction with other proteins which are crucial for the nuclear import of FBXO7. In support of this contention, we showed that the first 40 amino acids of FBXO7 isoform 1 are crucial for nuclear localization, as they efficiently direct profilin (a cytosolic protein) to the nucleus; however, the T22M mutation suppresses this effect. The C-terminus of the protein might also contain important motifs for nuclear import or export, as the overexpressed R498X mutant also displays an abnormal pattern of equally diffuse, cytosolic and nuclear localization.

Regarding the stability of the FBXO7 missense and truncated proteins encoded by the mutations causing PARK15, our data collectively suggest that the T22M, R378G and R498X mutants are all significantly unstable compared with the WT protein, at least in our overexpressing systems (Figure 8 and Figure S4). It is possible that the overexpressed FBXO7 mutants were insoluble and therefore not present in the cell lysate fraction. We therefore detected FBXO7 in insoluble fractions, but the amount of FBXO7 mutants was still lower than that of the WT counterpart (data not shown). Furthermore, formation of inclusions or membrane association of overexpressed FBXO7 was not observed. Last, we showed that the overexpressed WT and mutant FBXO7 are mainly degraded via the lysosomal pathway, with also a minor role of the proteasome pathway. Our data also suggest that the overexpressed R378G mutant is less stable than the WT FBXO7. The Iranian PARK15 patients might thus also suffer a severe depletion of the endogenous protein. It will be of interest to test this prediction in patients-derived cells, since we found that R378G does not significantly alter the nuclear localization in transfected cells. If sufficiently stable, this mutant protein could provide important clues about the nuclear function of FBXO7 that is lost in PARK15.

Last, we provide the initial characterization of the expression of the FBXO7 proteins in the normal human brain by immunohistochemistry. The nuclei of neurons throughout the cerebral cortex showed the strongest FBXO7 immunoreactivity, followed by the neurons in the subcortical diencephalic region, and the substantia nigra, while the lowest immunoreactivity was detected in the hippocampus and the cerebellar cortex (Figure 9 and data not shown).

Limited data on the expression of the *FBXO7* gene at mRNA level were reported in one of the studies that originally characterized this gene (named *FBX* therein) [14]. The FBXO7 mRNA appeared to be broadly expressed in human tissues, but enriched in bone marrow, liver, kidney, testis, and thyroid gland. Interestingly, a strong expression was also found in several regions of the human brain, such as the corpus callosum, caudate nucleus, substantia nigra, as well as in the spinal cord [14]. Furthermore, microarray data on the regional expression of the *FBXO7* gene in the human brain, are available in the ALLEN Human Brain Atlas website (http://www.brain-map.org, accessed on Dec. 1, 2010). In this Atlas, *FBXO7* expression levels are high in the cerebral cortex (particularly the frontal and parietal regions), the striatum, pallidum, thalamus, substantia nigra, red nucleus, and deep cerebellar nuclei, while low expression levels are documented in the hippocampus and the cerebellar cortex.

The *FBXO7* mRNA and proteins seem therefore highly expressed in the motor areas of the human brain, including both the extrapyramidal and the pyramidal systems, which fits with the clinical phenotype of parkinsonian-pyramidal disorder caused by the loss of the FBXO7 function in PARK15. Intriguingly, the widespread and abundant expression of the FBXO7 proteins in the frontal cerebral cortex suggests that additional, cognitive and behavioural disturbances of frontal type might be prominent in the phenotype. Interestingly, severe behavioural disturbances were noted in one of the Dutch PARK15 patients [10].

In conclusion, the common cellular abnormality found in the PARK15 patients from the Dutch and Italian families is the depletion of the FBXO7 isoform 1, which normally localizes mostly in the cell nucleus. The activity of FBXO7 in the nucleus appears therefore crucial for the maintenance of brain neurons and the pathogenesis of PARK15.

## Materials and Methods

### Ethics Statement

The study was approved by the *Medical Ethical Committee* (Medisch Ethische Toetsings Commissie, METC) of the Erasmus MC Rotterdam, and all participating subjects provided their informed consent.

### Subjects

The Dutch and Italian families with *FBXO7* mutations have been described previously by some of us [10].

### Cell culture and transfection

Lymphoblastoid cell lines were obtained by Epstein-Barr virus (EBV) immortalization of peripheral blood cells obtained from patients and controls, according to standard protocols. The lymphoblastoid cells were cultured in RPMI 1640 medium (Gibco) supplemented with 15% fetal calf serum, 50 U/ml penicillin and 50 mg/ml streptomycin, in a humidified 5% CO_2_ incubator. Human Embryonic Kidney (HEK) 293T cells, human neuroblastoma cells (SH-SY5Y), and human fibroblasts were grown in Dulbecco's modified Eagle's medium (DMEM) (Lonza). The HEK 293T cells were incubated in a humidified 5% CO_2_ incubator, and the SH-SY5Y cells and fibroblasts were incubated in a humidified 10% CO_2_ incubator. The HEK 293T cells were transiently transfected by polyethyleneimine (PEI, Polysciences Inc.). Lipofectamine™ LTX and PLUS™ Reagents (Invitrogen) transfection were used in SH-SY5Y cells according to the manufacturer's instructions. Furthermore, stable *FBXO7* knock down HEK 293T cells were generated using *FBXO7* shRNA (#TRCN 0000004339, Sigma); a non-targeting vector (shNT) (SHC002, Sigma) was used as control. Mouse primary hippocampal neurons were dissected and cultured as described previously [23]. After 20 days *in vitro*, the hippocampal neurons were transfected with constructs expressing wild type or mutant FBXO7 by using Lipofectamine 2000 (Invitrogen).

### *FBXO7* constructs

The full-length *FBXO7* cDNA (GenBank accession number NM_012179.3; NP_036311.3) was amplified by PCR using cDNA obtained from SH-SY5Y neuroblastoma cells as template. The PCR product was ligated into pcDNA™3.1/V5-His-TOPO (Invitrogen). After sequencing, the insert WT *FBXO7* was subcloned in frame into the mammalian expression plasmid pEGFP-C3 (BD biosciences), resulting in an N-terminal fusion protein eGFP-FBXO7. All primers used are given in the Supplementary Table S1. The untagged WT *FBXO7* was obtained using the QuikChange site-directed mutagenesis kit (Stratagene) by introducing a stop codon in front of the V5-His tag. All untagged *FBXO7* mutants (T22M, R378G, and R498X) were also prepared using the above-mentioned mutagenesis kit. The cDNA fragment encoding the first 40 amino acids of WT or T22M-mutant FBXO7 isoform 1 was amplified and subcloned in front of mCherry-labeled profilin (the mCherry-profilin construct was a gift from Josien Levenga, Clinical Genetics Department, Erasmus MC). The complete cDNA open reading frame of all constructs was verified by direct sequencing.

### Immunofluorescence

The HEK 293T and SH-SY5Y cells were seeded onto glass coverslips coated with 0.1% gelatin (Sigma-Aldrich), and transfected with untagged WT or mutant *FBXO7* constructs. The cells were fixed in 4% (w/v) paraformaldehyde in PBS for 10 minutes and permeabilized in 100% methanol for 20 minutes. The cells were then blocked with 3% bovine serum albumin (BSA, Sigma) in PBS (w/v) for 30 minutes, and probed at 4°C overnight with mouse polyclonal antibody raised against full-length FBXO7 (Abnova, 1/300). The samples were then incubated with Cy3-coupled secondary anti-mouse antibodies (Jackson ImmunoResearch, 1/200) for 1 hour. The cell nuclei were stained with Hoechst dye 33342 (Invitrogen), but the staining is shown in green color to increase image contrast. Cells were then mounted on slides with fluorescent mounting medium (DAKO). Fluorescence images were collected using a Leica SP5 confocal microscope (Leica Microsystems), and analyzed with the Leica Confocal Software.

Immunofluorescence of primary hippocampal neurons was performed 24 hours after transfection. The cells were fixed with 4% paraformaldehyde and permeabilized with staining buffer containing 50 mM Tris, 0.9% NaCl, 0.25% gelatin, and 0.5% Triton X-100, at pH 7.4. The subsequent antibody incubation was performed as described above.

### Quantitative PCR (qPCR)

Total RNA was isolated by RNA Bee (TEL-TEST Inc.) from EBV-transformed lymphoblastoid cells and converted into first-strand cDNA using the iScript™ cDNA Synthesis Kit (Bio-Rad). qPCR was carried out using a KAPA SYBR® FAST qPCR Kit (Kapa Biosystems) in the ABI Prism 7300 Sequence Detection System (Applied Biosystems). Thermal cycling conditions in the ABI Prism 7300 Detection System were as follows: denaturing step (95°C for 3 minutes), followed by 35 cycles of denaturing (95°C for 5 seconds), annealing and extension (60°C for 30 seconds). Fluorescence detection and data analysis were performed by ABI Prism 7300 SDS software (version 1.3.1, Applied Biosystems). Experiments were performed in triplicate using hypoxanthine phosphoribosyltransferase (HPRT) as the endogenous control for gene expression normalization.

The primers used for the qPCR studies, including one assay for the total *FBXO7* transcripts (isoform 1 and isoform 2), one assay specific for the isoform 1, and another assay specific for the isoform 2, are given in Supplementary Table S2.

### Western Blotting

The cells were washed with cold phosphate buffer (PBS) and harvested in lysis buffer containing 20mM Tris-HCl at pH 8.0, 150mM NaCl, 1mM NaVO_4_, 50mM NaF, 1% Triton X-100, and a protease inhibitor cocktail (Roche Molecular Biochemicals). The cells were lysed for 30 minutes on ice before centrifugation (15000 *g* for 10 minutes at 4°C), and then the total protein concentration of the supernatant was determined by using the bicinchoninic acid (BCA™) protein assay kit (Pierce) according to the manufacturer's instructions. The protein samples (40 micrograms) were separated by 6%–12% Criterion™ XT 4–12% Bis-Tris Gel (Bio-Rad), and then transferred to nitrocellulose membranes. Membranes were blocked with 5% low-fat milk powder (Fluka) in 1×PBS containing 0.1% Tween20 (PBST) for 1 hour at room temperature and incubated overnight at 4°C with mouse polyclonal antibody against full length FBXO7 (Abnova, 1/3000) and mouse monoclonal against actin (Abcam, 1/2500) or against eGFP (Roche, 1/2000). After washing 3 times with PBST, the membranes were incubated in the dark for 1 hour with PBST containing donkey anti-mouse secondary antibodies (800nm, LI-COR Biosciences Lincoln, 1/5000 dilution). After washing, the membranes were scanned using the Odyssey ™ Infrared Imager (Li-COR Biosciences). The integrated intensities of the protein bands were quantified by the Odyssey software.

### Proteasome and lysosomal-mediated degradation of FBXO7 mutants

The HEK 293T cells were seeded onto 6-well plates and transfected with 1.5 µg *FBXO7* constructs; empty vector pcDNA3 (Invitrogen, Carlsbad, CA, U.S.A.) was used as mock control. To test for the proteasomal degradation of the FBXO7 protein, 40 hours after transfection the cultured medium was replaced with DMEM containing either 42 µM MG-132 (Sigma-Aldrich) or vehicle control DMSO. After 6 hours incubation, the cells were harvested and analyzed by WB. The lysosomal degradation pathway was tested by adding 30mM NH_4_Cl to the cell culture 28 hours after transfection. The cells were incubated with NH_4_Cl for 20 hours and harvested for WB analysis.

### FBXO7 protein studies in human brain tissue

Human brain tissues were obtained from The Netherlands Brain Bank, Netherlands Institute for Neuroscience, Amsterdam. All material has been collected from donors from whom a written informed consent for brain autopsy and the use of the material and clinical information for research purposes had been obtained by the Netherlands Brain Bank. For immunohistochemistry, paraffin-embedded sections (7 µm) were analyzed from the brain of two non-parkinsonian, non-demented male donors (age at death 76 and 81 years-old). The following regions were studied: frontal, temporal, and occipital cerebral cortex, globus pallidum, hippocampus, substantia nigra, and cerebellum. Briefly, dewaxed sections were pretreated for antigen retrieval using pressure cooking in 0.1 M sodium citrate buffer (pH 6) for 5 min. Subsequently, sections were incubated overnight with antibodies against FBXO7 (Abnova, 1∶100) at 4°C. The broad spectrum poly–AP powervision reagent (immunoLogic) was used for 1 hour at room temperature for visualization. Enzyme detection was performed by using 1% new fuchsin, 1% sodiumnitrite, 0.03% naphtol AS-MX phosphate (Sigma), and 0.025% levamisol (Acros) for 20 minutes at room temperature. The sections were not counterstained but directly mounted in aquamount for examination under a light microscope.

### Data analysis and statistics

Quantitative data are expressed as means ± SEM based on at least three independent experiments. Statistical analyses were performed using contingency tables.

## Supporting Information

Figure S1 Validation of the specificity of the FBXO7 antibody by Western blotting in stable *FBXO7* gene knock down HEK 293T cells.
(PDF)

Figure S2 Validation of the specificity of the FBXO7 antibody by immunofluorescence in stable *FBXO7* gene knock down HEK 293T cells.
(PDF)

Figure S3 qPCR analysis of *FBXO7* isoform-specific transcripts in members of the PARK15 families and unrelated healthy controls (C1–C3).
(PDF)

Figure S4 Overexpression of wild type and mutant FBXO7.
(PDF)

Figure S5 Positively charged amino acids in the FBXO7 protein.
(PDF)

Table S1 Primers used for molecular cloning.
(PDF)

Table S2 Primers used for qPCR.
(PDF)
