# Supplementary material for: Loss of Nuclear Activity of the FBXO7 Protein in Patients with Parkinsonian-Pyramidal Syndrome (PARK15)
Source: PLoS One. 2011 Feb 11;6(2):e16983. doi: 10.1371/journal.pone.0016983 (PMC3037939; doi:10.1371/journal.pone.0016983)
Supplement: Figure S1 — Validation of the specificity of the FBXO7 antibody by Western blotting in stable FBXO7 gene knock down HEK 293T cells. (PDF) [file pone.0016983.s001.pdf]

Figure S1

Validation of the specificity of the FBXO7 antibody by Western blotting in stable *FBXO7* gene knock down HEK 293T cells

The FBXO7 protein is visualized by using a mouse anti-FBXO7 antibody (Abnova). Cells overexpressing the FBXO7 isoform 1 or isoform 2 are also shown as a reference. Actin is immunostained as loading control.

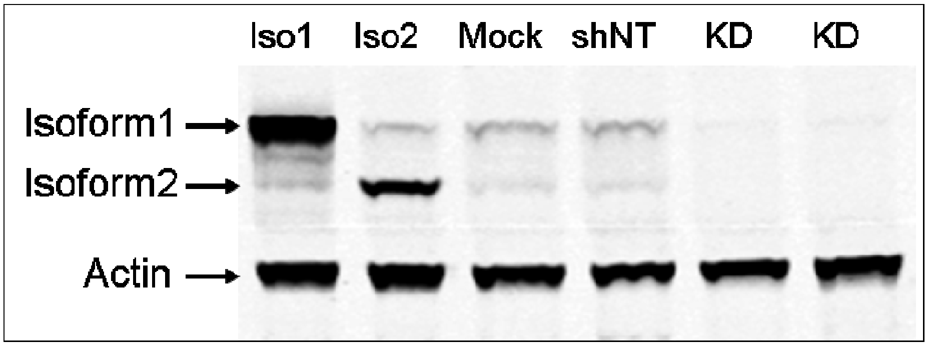

Iso1: cells transfected with FBXO7 isoform 1 expression construct
Iso2: cells transfected with FBXO7 isoform 2 expression construct
Mock: untransfected cells (endogenous FBXO7 proteins)
shNT: non-targeting shRNA
KD: *FBXO7* knock down shRNA
