# Supplementary material for: Loss of Nuclear Activity of the FBXO7 Protein in Patients with Parkinsonian-Pyramidal Syndrome (PARK15)
Source: PLoS One. 2011 Feb 11;6(2):e16983. doi: 10.1371/journal.pone.0016983 (PMC3037939; doi:10.1371/journal.pone.0016983)

Figure S2

Validation of the specificity of the FBXO7 antibody by immunofluorescence in stable *FBXO7* gene knock down HEK 293T cells

The endogenous FBXO7 protein is visualized in red by using a mouse primary anti-FBXO7 antibody (Abnova) and a Cy3-coupled secondary anti-mouse antibody. The nucleus (Hoechst staining) is depicted in green. (scale bars, 10 μm)

Mock: untransfected cells
shNT: non-targeting shRNA
KD: *FBXO7* knock down shRNA

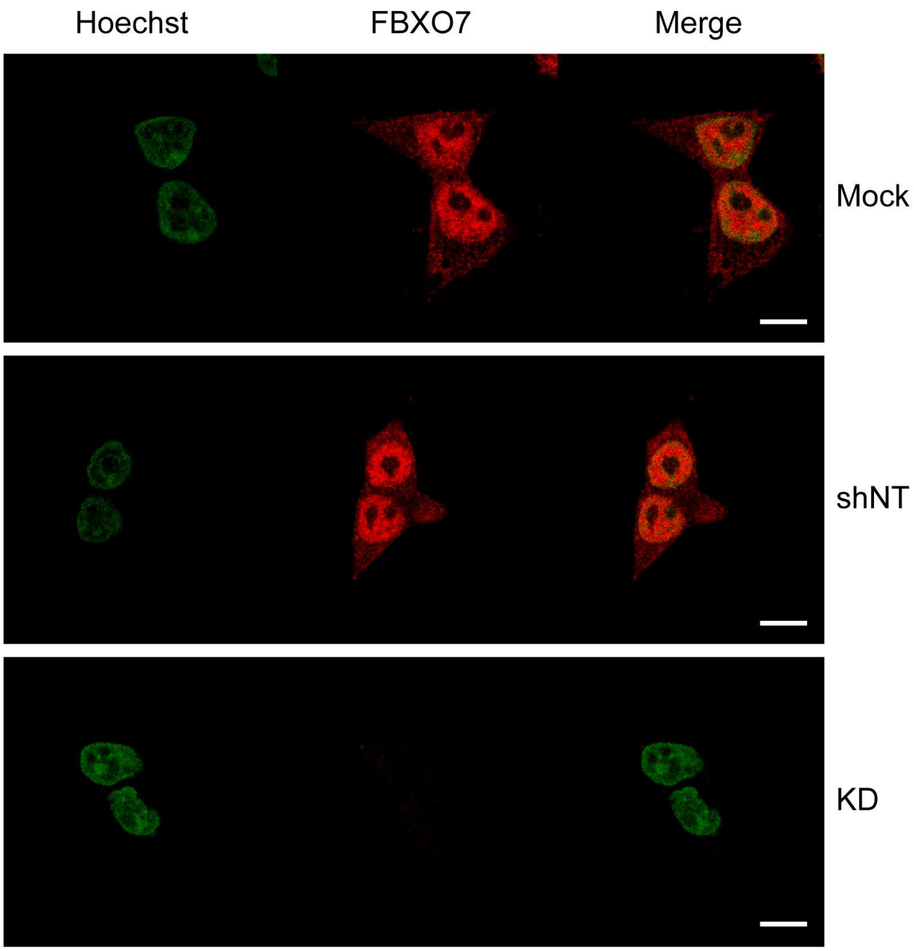

Supplement: Figure S2 — Validation of the specificity of the FBXO7 antibody by immunofluorescence in stable FBXO7 gene knock down HEK 293T cells. (PDF) [file pone.0016983.s002.pdf]
