# Supplementary material for: Loss of Nuclear Activity of the FBXO7 Protein in Patients with Parkinsonian-Pyramidal Syndrome (PARK15)
Source: PLoS One. 2011 Feb 11;6(2):e16983. doi: 10.1371/journal.pone.0016983 (PMC3037939; doi:10.1371/journal.pone.0016983)

## Figure S3

**qPCR analysis of *FBXO7* isoform-specific transcripts in members of the PARK15 families and unrelated healthy controls (C1-C3)**

**A – target transcript: *FBXO7* isoform 1; reference transcript: *HPRT***

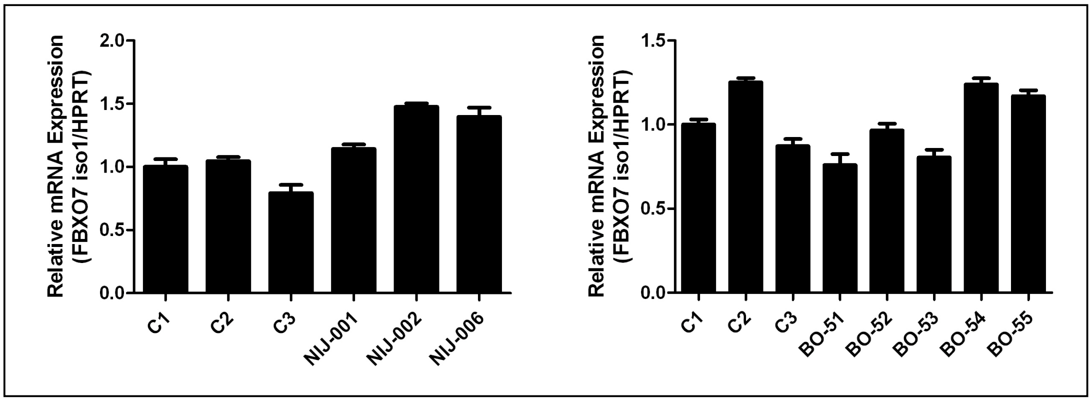


**B – target transcript: *FBXO7* isoform 2; reference transcript: *HPRT***

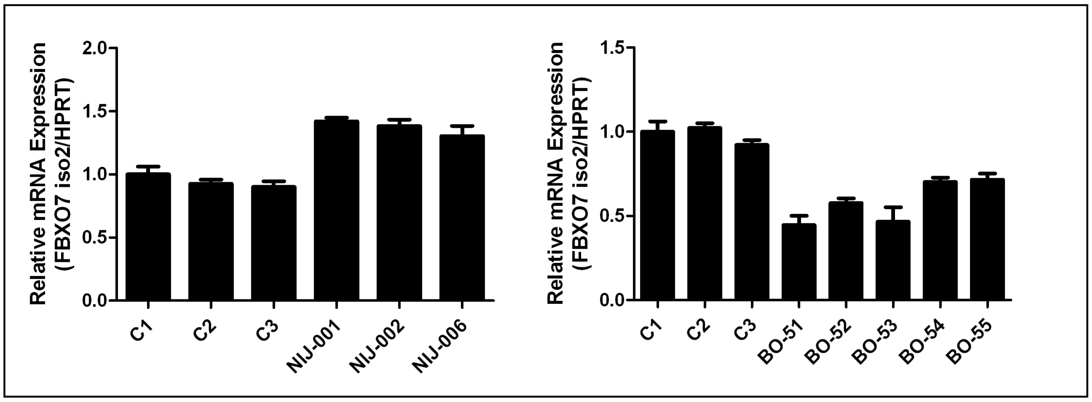


**C – target transcript: *FBXO7* isoform 1; reference transcript: *FBXO7* isoform 2**

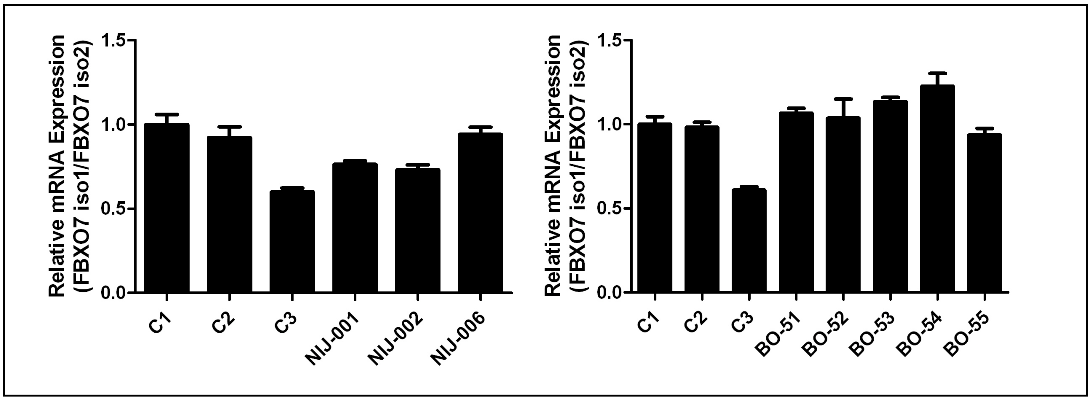

Supplement: Figure S3 — qPCR analysis of FBXO7 isoform-specific transcripts in members of the PARK15 families and unrelated healthy controls (C1–C3). (PDF) [file pone.0016983.s003.pdf]
