# Supplementary material for: Loss of Nuclear Activity of the FBXO7 Protein in Patients with Parkinsonian-Pyramidal Syndrome (PARK15)
Source: PLoS One. 2011 Feb 11;6(2):e16983. doi: 10.1371/journal.pone.0016983 (PMC3037939; doi:10.1371/journal.pone.0016983)

Figure S4 Overexpression of wild type and mutant FBXO7

Wild type and mutant FBXO7 isoform 1 proteins are co-transfected with eGFP in HEK 293T cells. The eGFP is used as control of transfection efficiency, while actin is used as loading control. The quantification of the protein levels is shown as FBXO7/actin and FBXO7/eGFP ratios (Odyssey software).

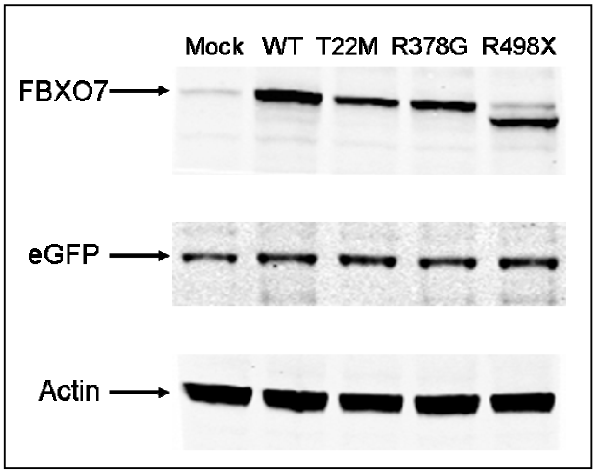

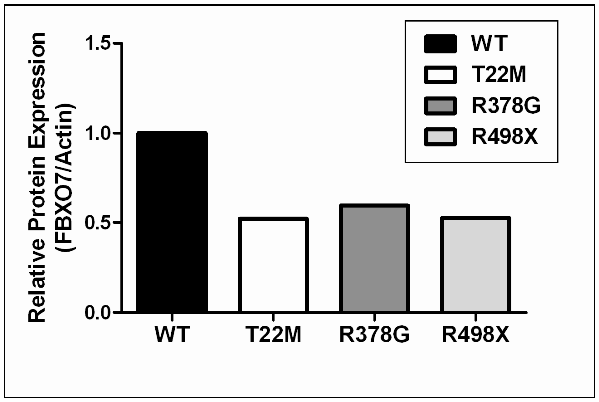

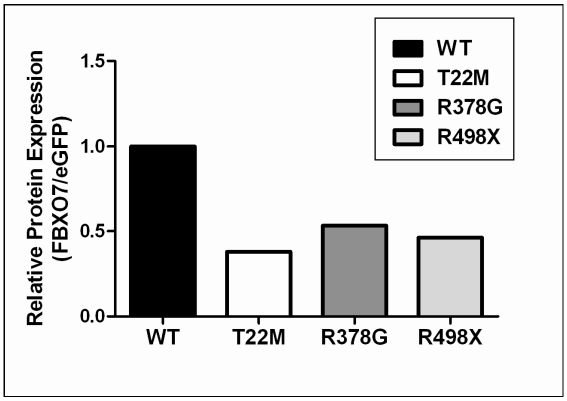

Supplement: Figure S4 — Overexpression of wild type and mutant FBXO7. (PDF) [file pone.0016983.s004.pdf]
