# Supplementary material for: Loss of Nuclear Activity of the FBXO7 Protein in Patients with Parkinsonian-Pyramidal Syndrome (PARK15)
Source: PLoS One. 2011 Feb 11;6(2):e16983. doi: 10.1371/journal.pone.0016983 (PMC3037939; doi:10.1371/journal.pone.0016983)
Supplement: Figure S5 — Positively charged amino acids in the FBXO7 protein. (PDF) [file pone.0016983.s005.pdf]

Figure S5 **Positively charged amino acids in the FBXO7 protein**

The positively charged amino acids in the FBXO7 protein are highlighted in yellow. Note the abundance at N-terminus.

**mrlrvrllkrtwplevpeteptlghlrshlrqsllctwgyssntrftitlnykdpltgdeetlasygivsgd
liclilqddipapnipsstdsehsslqnneqpslatssnqtsmqdeqpsdsfqgqaaqsgvwnd
dsmlgpsqnfeaesiqdnahmaegtgfypsepmlcsesvegqvphsletlyqsadcsdanda
livlihllmlesgyipqgteakalsmpekwklsgvyklqymhplcegssatltcvplgnlivvnatlki
nneirsvkrlqllpesfickeklgenvaniykdlqklsrlfkdqlvypllaftrqalnlpdvfglvvlplelk
lrifrlldvrsvlslsavcrdlftasndpllwrflylrdfrdntvrvqdtdwkelyrkrhiqrkespkgrfv
mllpssthtipfypnplhprpfpssrlppgiiggeydqrptlpyvgdpisslipgpgetpsqfpplrpr
fdpvgplpgpnpilpgrggpndrfpfrpsrgrptdgrlsfm**
