# Supplementary material for: Loss of Nuclear Activity of the FBXO7 Protein in Patients with Parkinsonian-Pyramidal Syndrome (PARK15)
Source: PLoS One. 2011 Feb 11;6(2):e16983. doi: 10.1371/journal.pone.0016983 (PMC3037939; doi:10.1371/journal.pone.0016983)
Supplement: Table S1 — Primers used for molecular cloning. (PDF) [file pone.0016983.s006.pdf]

Table S1 Primers used for molecular cloning

Constructs	Forward primer (5’-3’)	Reverse primer (5’-3’)
FBXO7-V5-His	TCGCCAGTCCGGGGTCGTC	AAGCTTCATGAATGACAGCCGGCCATC
eGFP-FBXO7	CAGGAGAAGCTTAGGCTGCGGGTGCG	CGCCCGGGCTTCATGAATGACAGC
WT FBXO7	GCCGGCTGTCATTCATGTAGCTTAAGGGCAATTCTGC	GCAGAATTGCCCTTAAGCTACATGAATGACAGCCGGC
T22M FBXO7	CCCGAGACGGAGCCGATGCTGGGGCATTTGCGC	GCGCAAATGCCCCAGCATCGGCTCCGTCTCGGG
R378G FBXO7	GGAGGTTTTTATATCTGGGTGATTTTCGAGAC	GTCTCGAAAATCACCCAGATATAAAAACCTCC
R498X FBXO7	CCTAACCCCATCTTGCCAGGGTGAGGCGGCCC	GGGCCGCCTCACCCTGGCAAGATGGGGTTAGG
FBXO7-profilin	GCCAGCTAGCATGAGGCTGCGG	GCAGGCTAGCGTGTACCCCCAG
