# Supplementary material for: Loss of Nuclear Activity of the FBXO7 Protein in Patients with Parkinsonian-Pyramidal Syndrome (PARK15)
Source: PLoS One. 2011 Feb 11;6(2):e16983. doi: 10.1371/journal.pone.0016983 (PMC3037939; doi:10.1371/journal.pone.0016983)
Supplement: Table S2 — Primers used for qPCR. (PDF) [file pone.0016983.s007.pdf]

<u>Table S2</u> Primers used for qPCR

Gene	Forward primer (5'-3')	Position	Reverse primer (5'-3')	Position
FBXO7 transcript 1	AGTCCCTGCTGTGCACCTG	Exon 1a	CGCTGGAATGTCATCTTGAAGA	Exon 2a
FBXO7 transcript 2	AACATGGCCCGGCCTC	Exon 1b	TTCTGGAGTGAAGAATGCTCTGAA	Exon 2b
FBXO 7 **Transcript 1 and 2**	GTCTGCGGTTTGTCGTGACC	Exon 7	TCTTGAACTCTGACAGTATTG	Exon 8
HPRT	TGACACTGGCAAAACAATGCA		GGTCCTTTTCACCAGCAAGCT	
